# Centriolin interacts with HectD1 in a cell cycle dependent manner

**DOI:** 10.1186/s13104-023-06670-y

**Published:** 2023-12-19

**Authors:** Jesus Salas, Alexander Garcia, Vancy Zora, Sean Dornbush, Fady Mousa-Ibrahim, Hanna Fogg, Zeynep Gromley, Adam Gromley

**Affiliations:** DeBusk College of Osteopathic Medicine, Lincoln Memorial University, Harrogate, TN USA

**Keywords:** HECTD1, Centriolin, Centrosome, Mitosis

## Abstract

**Objective:**

The centrosome is universally recognized as the microtubule organizing center of animal cells, but emerging evidence suggests that it has other important functions including primary cilia formation, DNA damage checkpoints, and cell cycle progression. Despite this, the role of individual components of the centrosome remains unclear. Previous studies suggest that one component, centriolin, has an important function in cytokinesis and cell cycle progression, although its exact role in these processes is not known. To determine how centriolin influences the progression through the cell cycle, we sought to identify interacting partners that may be involved in regulating its function.

**Results:**

This study provides evidence that the ubiquitin E3 ligase HectD1 binds to centriolin and that this association likely accounts for our observation that HectD1 co-localizes with centriolin at the centrosome during mitosis. In addition to its centrosomal localization, we also show that the expression of HectD1 fluctuates throughout the cell cycle, with the highest levels during mitosis, coinciding with a marked reduction in centriolin expression. We propose that the interaction between HectD1 and centriolin may be necessary for normal cell cycle progression and we speculate that this function may involve HectD1-mediated degradation of centriolin.

**Supplementary Information:**

The online version contains supplementary material available at 10.1186/s13104-023-06670-y.

## Introduction

The primary microtubule organizing center in animal cells is the centrosome. Located adjacent to the nucleus in interphase cells, this organelle is responsible for organizing the elaborate microtubule array that serves as an avenue for the intracellular trafficking events of interphase. During S phase, the centrosome duplicates and later separates to form the poles of the mitotic spindle, thus being responsible for proper segregation of the chromosomes in mitosis. In addition to these universally recognized roles in microtubule organization [1], there are reports of individual protein components of the centrosome having essential roles in other cellular processes such as cell cycle progression [2–4], mitotic exit [1, 2, 5], primary cilia formation [6], cytokinesis [5, 7], and DNA damage checkpoints [1].

Structurally, the centrosome is composed of a pair of orthogonally oriented microtubule barrels, known as centrioles, surrounded by a protein complex called the pericentriolar material [1]. Within the pericentriolar material, pericentrin and gamma tubulin form a lattice-like structure that is responsible for the nucleation of microtubules. Other components of the pericentriolar material mediate the anchoring of microtubules. These include PCM1, CEP192, and CDK5RAP2 [8].

Protein components of the centrioles include ninein, centrin, Odf2/cenexin, CEP170, and centriolin [8]. Interestingly, centriolin has been shown to have multiple roles in cell division independent of microtubule organization. This protein was initially found to be necessary for mitotic exit, as its disruption using siRNA [2] and CRISPR/Cas9 [9] results in dysfunctional cell division and arrest of the cell in the G1 phase of the cell cycle. Additional studies determined that centriolin plays a key role in the final abscission event at the end of cytokinesis, acting as a scaffold at the midbody for the recruitment of complexes that control the membrane fusion events necessary for physical separation of the daughter cells [7]. Although these studies have brought insights into the role of centriolin in cell cycle progression, it is unclear if interactions with other proteins are necessary for these functions.

Successful cell cycle progression requires a concerted program of post-translational modifications, including ubiquitination, of key proteins at specific stages of the cell cycle. A family of ubiquitinating enzymes that has been implicated in the regulation of the cell cycle is the Hect E3 family of E3 ligases [10]. One member of this family, HectD1, originally known as EULIR, was first identified as being responsible for the ubiquitination of the inhibin B receptor [11]. Subsequently, studies have shown that HectD1’s function is necessary for a variety of cellular and developmental processes including cell migration [12], adhesion [11], normal formation of the placenta [13], and neural tube closure during embryonic development [14]. It has also been proposed that HectD1 function may play an important part in the control of the cell cycle, although its exact role remains unclear [10]. Mechanistically, HectD1 is known to attached K63-linked and K48-linked ubiquitin chains to its substrate proteins [15]. In contrast to K48 linkages, which are responsible for targeting the substrate to the proteosome for degradation, K63 ubiquitination regulates a variety of processes including intracellular trafficking and protein localization [16].

In this paper, we describe a novel interaction between the E3 ligase HectD1 and the centrosome protein centriolin. This interaction was initially discovered in a yeast two hybrid screen designed to reveal centriolin binding partners. To confirm this interaction in mammalian cells, immunofluorescence microscopy was used in cultured Hela cells to demonstrate a colocalization of these proteins at the spindle poles (centrosomes) during mitosis, and co-immunoprecipitation experiments confirm a physical interaction in synchronized mitotic cells. It was also observed that the expression levels of HectD1 appear to peak during mitosis, coinciding with a perceivable reduction in centriolin levels. Although the significance of this interaction is currently unknown, future studies will attempt to determine if centriolin serves as a substrate for the ubiquitin ligase activity of HectD1 and if this ubiquitination directly leads to centriolin’s degradation.

## Materials and methods

### Cell culture

HeLa cells (cat # CCL-2, ATCC) were maintained in DMEM medium (cat # 10013CV, Corning) supplemented with 10% FBS (cat # 35010CV, Corning), and penicillin–streptomycin-glutamine (cat # 10378016, Gibco) in a humidified incubator at 5% CO_2_ and 37 °C. Synchronization studies were carried out as described [17]. Cells were synchronized in G0/G1 by serum starvation. S-phase synchronization was achieved with a double thymidine block, and nocodazole treatment followed by mechanical shake-off was used to collect M-phase cells.

### Antibodies and immunofluorescence

The following primary antibodies were used for immunofluorescence staining: centriolin (Thermo Fisher, cat # PA5-54219), α-tubulin (Invitrogen, cat. # 62204), and HectD1 (cat # sc-517169, Santa Cruz Biotechnology). Cells were fixed in methanol and processed for immunofluorescence as described [18], using Alexa Fluor conjugated secondary antibodies ( Thermo Fisher, cat# A-21202 and A-11036) for immunofluorescence detection. All immunofluorescence images were captured on a Nikon Eclipse 50i immunofluorescence microscope equipped with a Nikon DXM1200C digital camera and a 40 × Plan Fluor objective with a 0.75 numerical aperture.

### Yeast two-hybrid analysis

A fragment containing amino acids 1–250 from the N-terminus of human centriolin (Genbank accession # KAI4008249) was cloned into EcoRI and SalI sites of the pGBKT7 vector and used as bait in the Matchmaker Gold Yeast Two-Hybrid System (Clontech, cat# 630489) per the manufacturer’s instructions. A screen for interactions against a universal normalized human cDNA expression library was performed (Clontech, cat# 630481). Positive clones were sequenced and the identity of each was determined by NCBI BLAST search (https://blast.ncbi.nlm.nih.gov/Blast.cgi).

### Immunoprecipitations and western blotting

Hela cell lysates were prepared using RIPA buffer (MilliporeSigma, cat# 20188) containing protease inhibitors (Thermo Fisher, cat# A32953) and western blotting was performed using standard procedures [19]. Primary antibodies for centriolin (Thermo Fisher, cat # PA5-54219), α-tubulin (Invitrogen, cat. # 62204), and HectD1 (cat # sc-517169, Santa Cruz Biotechnology), and secondary HRP-conjugated antibodies (Thermo Fisher, cat# 31466 and 32430) were used. For immunoprecipitations, antibodies to centriolin were added to Hela cell extracts and incubated at 4 °C overnight. Samples were then incubated with protein A/G beads (Thermo Scientific, cat# 20423) at 4 °C for 2 h, followed by SDS-PAGE and immunoblotting [20].

## Results

### Yeast two hybrid and immunoprecipitations reveal a direct interaction between centriolin and HectD1

Centriolin, an integral component of the centrosome, plays important roles in various cellular processes, including cell cycle progression and cytokinesis [7]. Structurally, centriolin is a large protein containing 2325 amino acids with several predicted coiled-coil regions interrupted by non-coiled domains [2]. In addition, the first 250 amino acids of the N-terminus contain multiple leucine-rich repeats (LRRs) that have been shown to be necessary for the successful completion of cell division [2, 7].

**Fig. 1:**
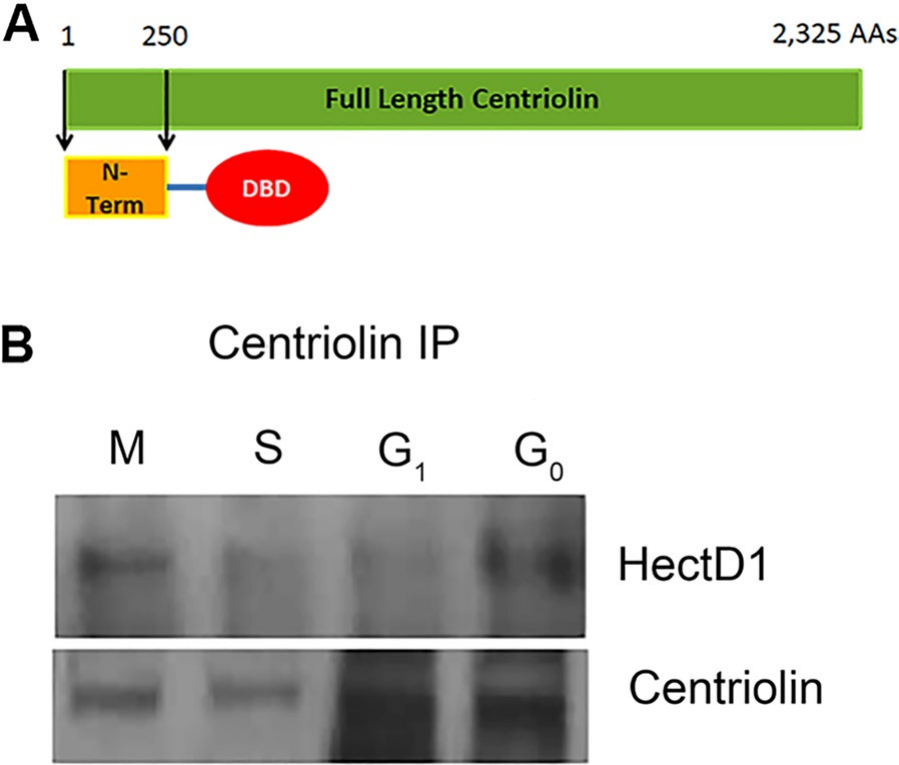
Yeast two hybrid and immunoprecipitations reveal a direct interaction between centriolin and HectD1. **A** Schematic shows the N-terminal region (amino acids 1–250) of the centriolin protein that was fused to the DNA binding domain (DBD) of the pGBKT7 vector. This construct was used in a yeast two hybrid screen that revealed an interaction between centriolin and HectD1. **B** Hela cells were arrested in the indicated cell cycle stages and cell lysates were subjected to immunoprecipitation using antibodies for centriolin. Western blot was then performed on immunoprecipitates using either HectD1 antibodies or centriolin antibodies

Due to the large number of predicted binding domains within the centriolin sequence, it is likely that this protein has multiple binding partners in the cell, which could influence its functions throughout the cell cycle. In order to identify any potential protein interactions, a yeast two hybrid screen was performed. This screen used the first 250 amino acids of the N-terminus of centriolin, containing the leucine rich repeat regions (Fig. 1A), as bait in the Clontech Matchmaker Gold Yeast Two-Hybrid System to identify interacting proteins in a normalized human cDNA expression library. This screen produced several positive clones, with a majority being Golgi- and ubiquitin-associated proteins. One of these ubiquitin-associated proteins was HectD1.

HectD1 is a member of the Hect family of ubiquitin E3 ligases, which are responsible for adding polyubiquitin tags to their target proteins. The function of these tags depends on the type of linkage between the ubiquitin molecules. K48-linked polyubiquitin chains are known to mark proteins for degradation in the proteosome, whereas K63-linked chains can be used to change the subcellular localization of the protein. Previous studies have shown that, under different conditions, HectD1 can mediate either of these linkages [10].

Because centriolin has essential roles in cell cycle progression and mitosis, we sought to confirm its interaction with HectD1 in cultured human cells and to determine if this interaction is cell cycle dependent. Hela cells were synchronized in G0, G1, S, and M phases using established protocols [17]. Lysates from these synchronized cells were immunoprecipitated using antibodies that specifically recognize centriolin. Western blot was then performed to determine if HectD1 was co-immunoprecipitated in these samples (Fig. 1B). As shown, a perceptible amount of HectD1 was co-immunoprecipitated with centriolin in all phases of the cell cycle. Interestingly, the highest levels of co-immunoprecipitation occurred in the mitotic cells. This data suggests that a low level of interaction may exist between centriolin and HectD1 throughout the cell cycle, but this interaction appears to increase precipitously during mitosis.

### Centriolin and HectD1 co-localize at the spindle poles in mitosis

**Fig. 2:**
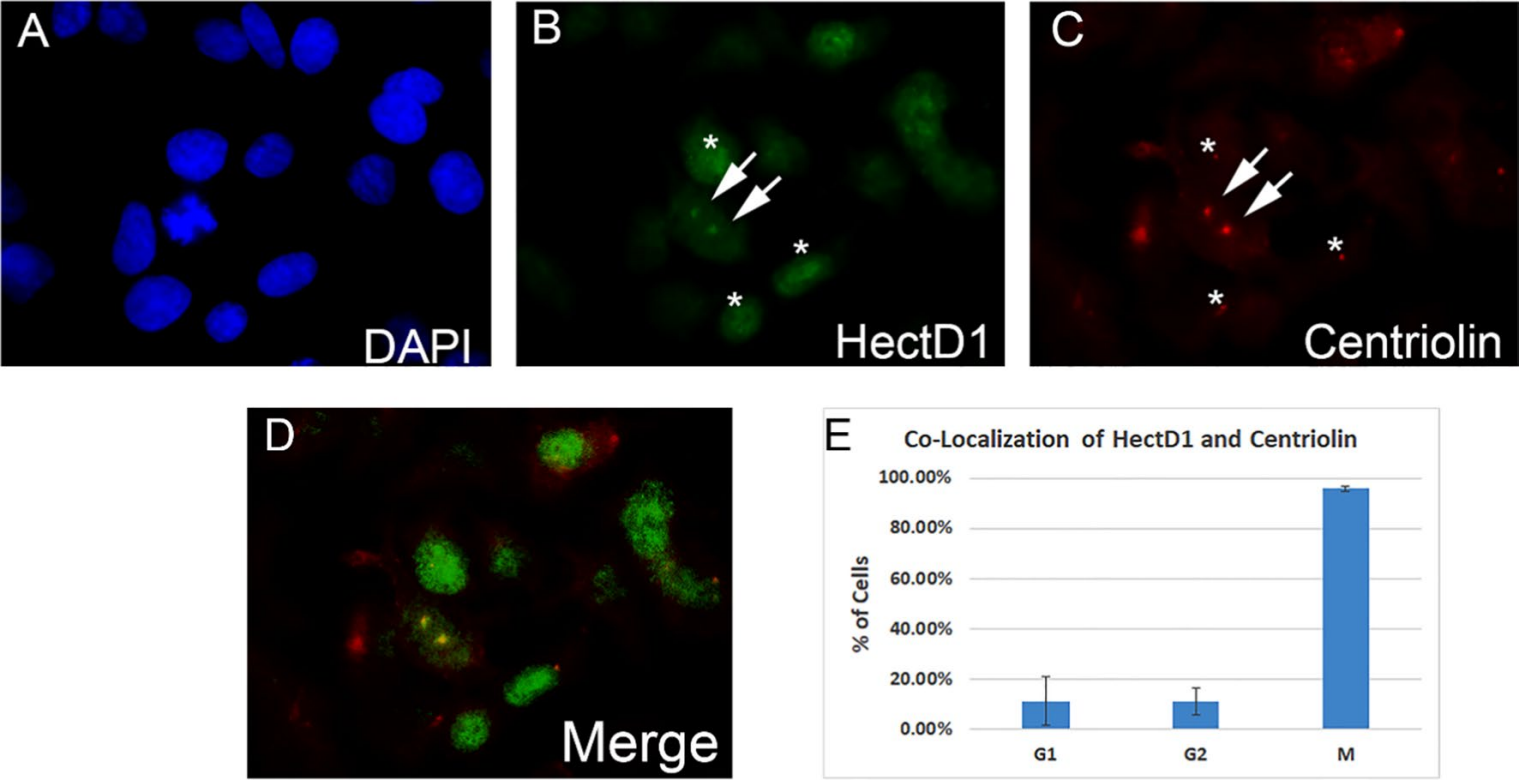
Centriolin and HectD1 co-localize at the spindle poles in mitosis. Immunofluorescence staining was performed on an asynchronous population of Hela cells. DAPI stain (blue, **A**)  indicates the nuclei of individual cells and also reveals mitotic events. Antibodies against human HectD1 protein (green, **B**) and human centriolin protein (red, **C**) were used. Note that colocalization is present only in the cell undergoing mitosis (arrows), whereas no colocalization can be seen in interphase cells (asterisks). Colocalization is also shown in the Merge image (panel **D**), with quantitation in panel **E** (n = 3)

Although our co-immunoprecipitation results suggest that there is a robust interaction between centriolin and HectD1 during the mitotic phase of the cell cycle, the location of this interaction within the cell was not apparent from these studies. In order to identify the subcellular localization of these proteins in intact cells, immunofluorescence staining was carried out on an asynchronous population of Hela cells. Our results revealed a specific co-localization of centriolin and HectD1 at the spindle poles (centrosomes) of mitotic cells (Fig. 2, arrows). In contrast, there did not appear to be any discernable co-localization of these two proteins during the other phases of the cell cycle (Fig. 2, asterisks).

### The expression of centriolin and HectD1 fluctuate throughout the cell cycle

**Fig. 3:**
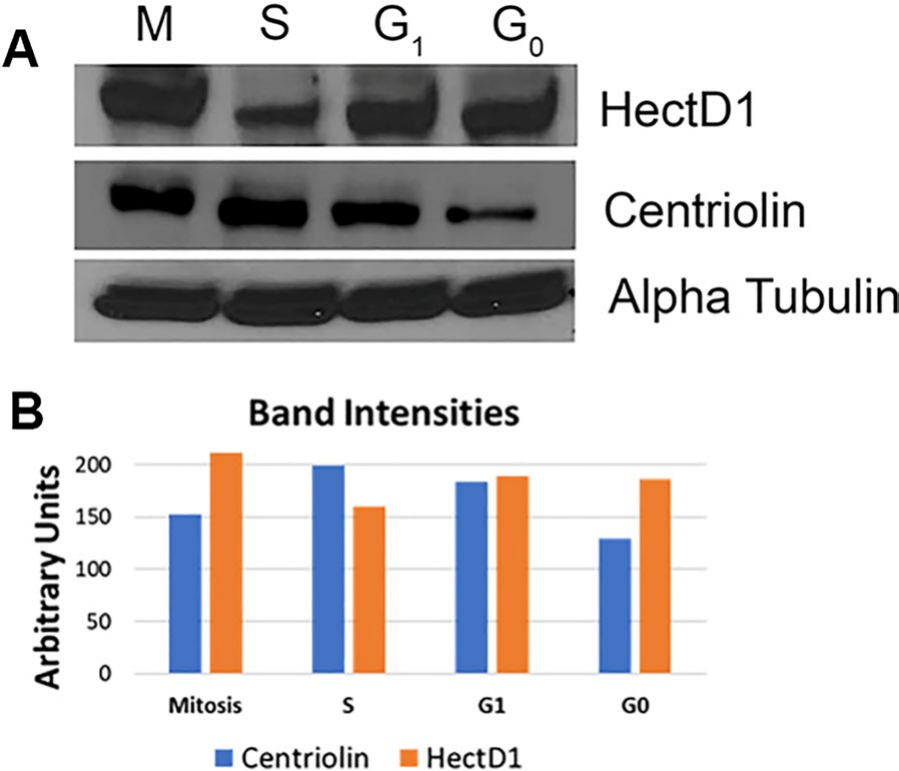
The expression of centriolin and HectD1 fluctuate throughout the cell cycle. Hela cells were arrested in the indicated cell cycle stages and western blot was performed on the cell lysates. HectD1 levels remain relatively constant in G0/G1, but fall during S phase, followed by a dramatic increase in mitosis. In contrast, centriolin displays a steady increase in expression from G0/G1 to S phase but drops in mitosis. Alpha tubulin was used as a loading control

In an attempt to further clarify the dynamics of centriolin and HectD1 expression throughout the cell cycle, we performed western blot on our synchronized Hela cell lysates. Interestingly, it was found that the amount of centriolin protein increased from G0 to S phase, but began to wane as the cells entered mitosis. In contrast, HectD1 expression appeared to be inversely proportional to that of centriolin, with high expression during G0 and G1, and lowest in S phase, when centriolin expression is highest (Fig. 3) (Additional file 1). Interestingly, HectD1 levels appear to rise again in mitosis, reaching its highest levels at this time, with a coordinate drop in centriolin levels. Although the significance of this disparity in expression between these two proteins is currently unknown, it is possible that centriolin may be serving as a substrate for HectD1 during mitosis.

## Discussion

Due to the relatively large size of centriolin and the presence of multiple coiled-coil regions within its amino acid sequence, it has been hypothesized that centriolin may function as a scaffold, having the ability to bind to a variety of different proteins with varying functions. In fact, our data provide support to this hypothesis, as our yeast two hybrid screen revealed several putative interacting partners with varying cellular functions. Many of these proteins are associated with the Golgi and ubiquitin, but a few are components of cellular signaling pathways. We are currently validating the interactions between centriolin and these other proteins in Hela cell extracts.

The cell cycle dependent interactions between centriolin and HectD1 are intriguing. To our knowledge, this is the first report of centriolin interactions with a Hect family E3 ligase. Our data suggest that HectD1 may be acting on centriolin to coordinate progression through the cell cycle and subsequent exit from mitosis. In fact, centriolin has been shown to have sequence homology to members of the mitotic exit network (MEN) in yeast, and elimination of centriolin protein has been reported to cause a disruption in the transition between mitosis and the subsequent G1 phase following division (2, 9). Furthermore, our data show that HectD1 levels are highest in mitosis, at a time when centriolin is reduced. This presents the intriguing possibility that HectD1 may be involved in the control of mitotic progression via ubiquitin-induced degradation of centriolin. In fact, the N-terminal domain of centriolin that was used in our yeast two hybrid screen contains predicted ubiquitination motifs. Further studies are needed to verify these putative ubiquitination sites and to determine if centriolin is indeed degraded via cell cycle dependent, HectD1-mediated ubiqtuitination.

## Limitations

A limitation of our studies is that they were conducted only in Hela cell cultures. It would be important to repeat these experiments in other transformed cell lines, as well as primary cell cultures, to confirm the effects are consistent in other cell types.

### Supplementary Information

**Additional file 1: Figure S1.** Full blots for Figure 1B. Left, Centriolin IP, HectD1 probe. Right, Centriolin IP, Centriolin probe. The cropped bands for Centriolin and HectD1 that were used to generate Figure 1B are indicated by the red boxes. Panel C is the Alpha Tubulin blot from this experiment showing equal amounts of total protein were present. **Figure S2.** Full blots for Fig. 3A. Cropped bands for Centriolin, HectD1, and Alpha Tubulin used to generate Fig. 3A are indicated by the red boxes.

## Data Availability

The datasets supporting the conclusions of this article are included within the article.

## Abbreviation

RIPA: Radioimmunoprecipitation Assay
DNA: Deoxyribonucleic Acid
siRNA: small interfering RNA
CRISPR: Clustered Regularly Interspaced Short Palindromic Repeats
EULIR: E3 Ubiquitin Ligase for Inhibin Receptor
ATCC: American Type Culture Collection
DMEM: Dulbecco's Modified Eagle Medium
SDS-PAGE: Sodium Dodecyl Sulfate Polyacrylamide Gel Electrophoresis
