# Supplementary material for: Centriolin interacts with HectD1 in a cell cycle dependent manner
Source: BMC Res Notes. 2023 Dec 19;16:375. doi: 10.1186/s13104-023-06670-y (PMC10731774; doi:10.1186/s13104-023-06670-y)
Supplement: Supplementary file 1 — Additional file 1: Figure S1. Full blots for Figure 1B. Left, Centriolin IP, HectD1 probe. Right, Centriolin IP, Centriolin probe. The cropped bands for Centriolin and HectD1 that were used to generate Figure 1B are indicated by the red boxes. Panel C is the Alpha Tubulin blot from this experiment showing equal amounts of total protein were present. Figure S2. Full blots for Fig. 3A. Cropped bands for Centriolin, HectD1, and Alpha Tubulin used to generate Fig. 3A are indicated by the red boxes. [file 13104_2023_6670_MOESM1_ESM.docx]

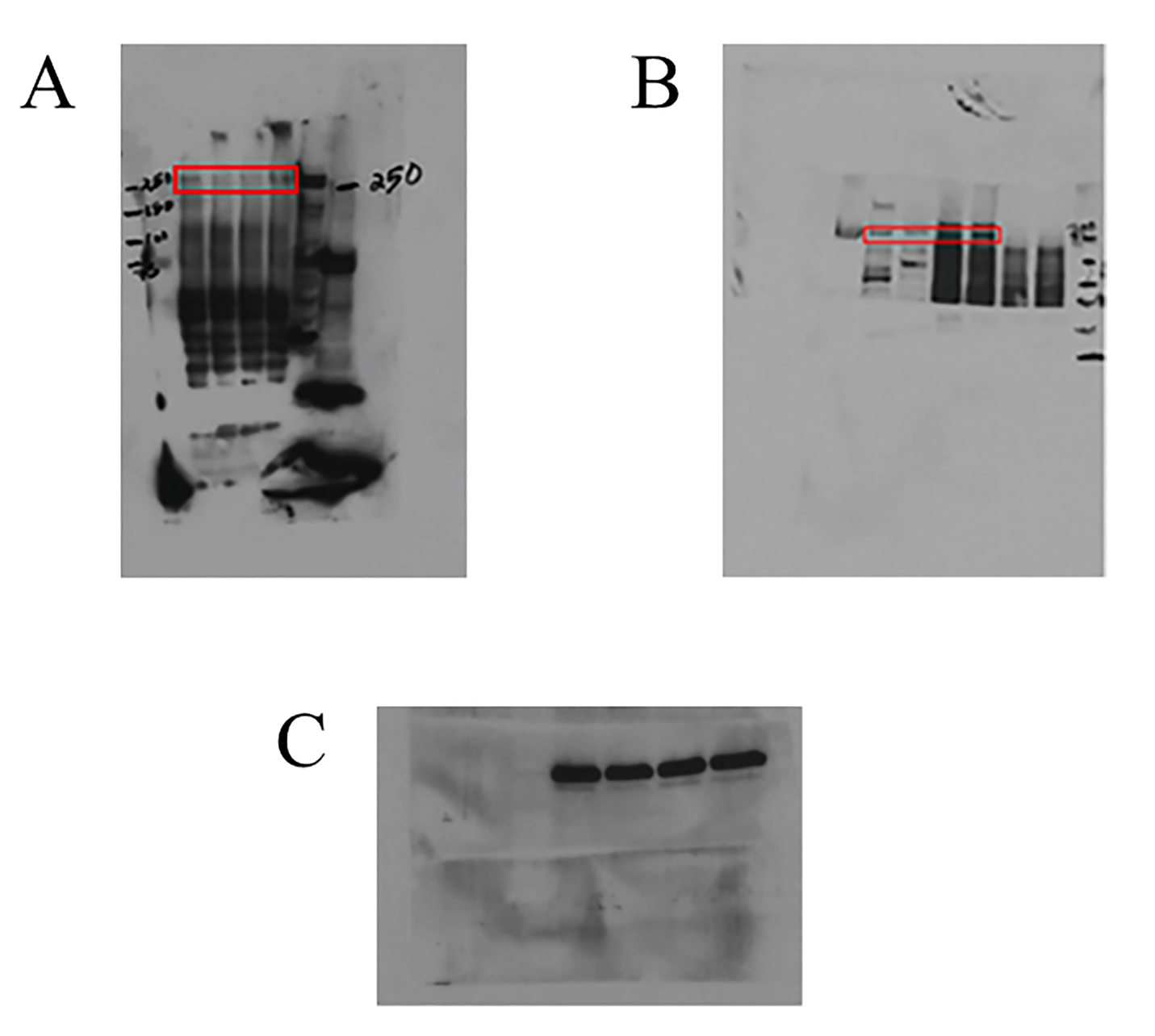


Additional file 1: Figure S1. Full blots for Figure 1B. Left, Centriolin IP, HectD1 probe. Right, Centriolin IP, Centriolin probe. The cropped bands for Centriolin and HectD1 that were used to generate Figure 1B are indicated by the red boxes. Panel C is the Alpha Tubulin blot from this experiment showing equal amounts of total protein were present.


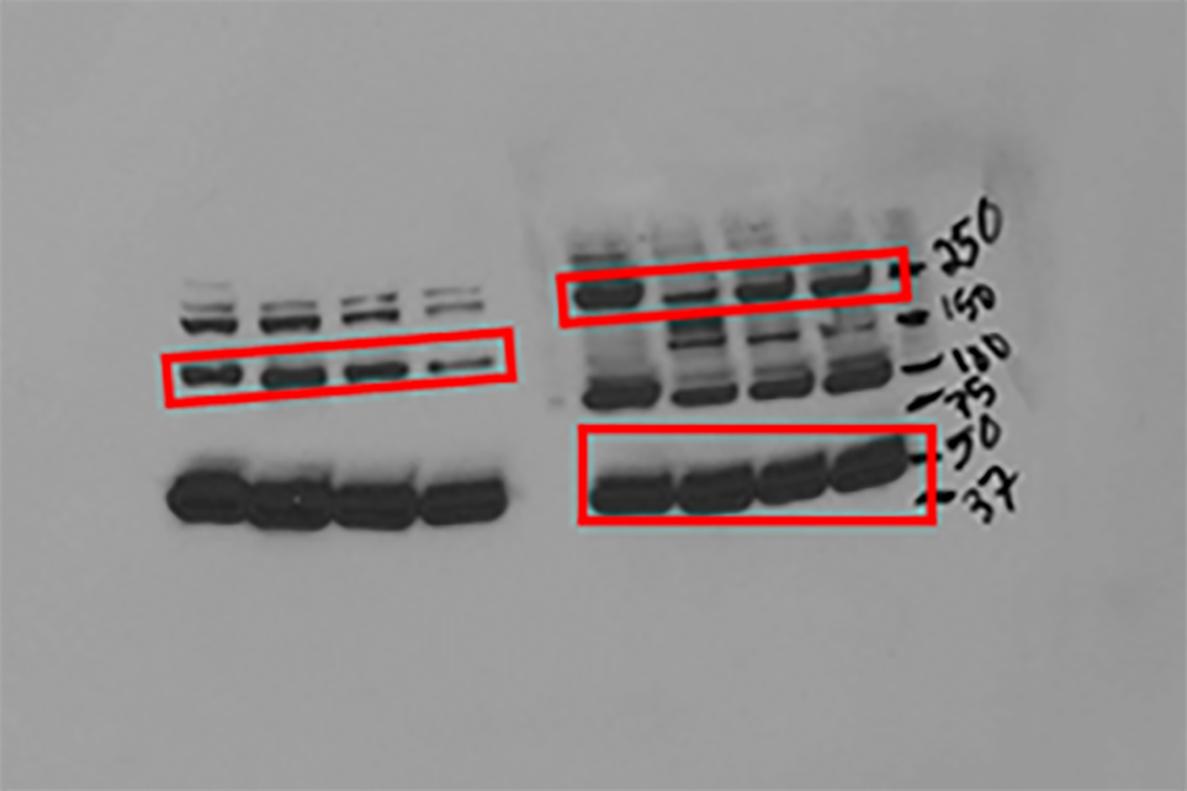


Additional file 1: Figure S2. Full blots for Fig 3A. Cropped bands for Centriolin, HectD1, and Alpha Tubulin used to generate figure 3A are indicated by the red boxes.
